# A new tool to evaluate postgraduate training posts: the Job Evaluation Survey Tool (JEST)

**DOI:** 10.1186/1472-6920-14-210

**Published:** 2014-10-02

**Authors:** David Wall, Helen Goodyear, Baldev Singh, Andrew Whitehouse, Elizabeth Hughes, Jonathan Howes

**Affiliations:** Health Education West Midlands, St Chad’s Court, 213 Hagley Road, Birmingham, B16 9RG UK

**Keywords:** Evaluation of training, Quality management, Quality assurance, Training posts

## Abstract

**Background:**

Three reports in 2013 about healthcare and patient safety in the UK, namely Berwick, Francis and Keogh have highlighted the need for junior doctors’ views about their training experience to be heard. In the UK, the General Medical Council (GMC) quality assures medical training programmes and requires postgraduate deaneries to undertake quality management and monitoring of all training posts in their area. The aim of this study was to develop a simple trainee questionnaire for evaluation of postgraduate training posts based on the GMC, UK standards and to look at the reliability and validity including comparison with a well-established and internationally validated tool, the Postgraduate Hospital Educational Environment Measure (PHEEM).

**Methods:**

The Job Evaluation Survey Tool (JEST), a fifteen item job evaluation questionnaire was drawn up in 2006, piloted with Foundation doctors (2007), field tested with specialist paediatric registrars (2008) and used over a three year period (2008–11) by Foundation Doctors. Statistical analyses including descriptives, reliability, correlation and factor analysis were undertaken and JEST compared with PHEEM.

**Results:**

The JEST had a reliability of 0.91 in the pilot study of 76 Foundation doctors, 0.88 in field testing of 173 Paediatric specialist registrars and 0.91 in three years of general use in foundation training with 3367 doctors completing JEST. Correlation of JEST with PHEEM was 0.80 (p < 0.001). Factor analysis showed two factors, a teaching factor and a social and lifestyle one.

**Conclusion:**

The JEST has proved to be a simple, valid and reliable evaluation tool in the monitoring and evaluation of postgraduate hospital training posts.

## Background

Three UK reports, published in 2013, looking at quality and safety of care in the NHS have highlighted the need for trainees’ views about their training experiences to be heard [1–3]. Junior doctors have been described as powerful agents for change [4] and the eyes and ears of the NHS [1]. The Francis report had 290 recommendations for improvement including 21 for education and training [1]. These stated that Postgraduate Deans should ensure an effective programme of monitoring and advised the use of trainee surveys especially as a source of information on patient safety [1].

The importance of quality assurance (QA) and quality improvement in medical education is accepted worldwide. The World Federation for Medical Education agrees standards for postgraduate medical education [5, 6]. Each country has its own QA process with some training programmes being accredited by Royal Colleges such as in Canada and others such as USA, Australia and UK by councils (Accreditation Council for Graduate Medical Education, Australian Medical Council and General Medical Council (GMC) respectively). A variety of methods have been used to evaluate posts including analysis of annual reports and site visits. Some countries include a trainee survey for both interns (first year of postgraduate training) and residents (specialty trainees) e.g. USA, Canada and UK [7, 8]. Post review needs to be a robust ongoing process which ensures that training is taking place in a supportive and constructive educational environment with good educational practice. There have been a number of tools developed to look at educational environment or climate summarised as what is encouraged, rewarded, emphasised and ways of working that are expressed [9]. These include the Postgraduate Hospital Educational environment measure (PHEEM) developed in the UK, a 40 item questionnaire [10] and Dutch Residency Educational Climate Test (D-RECT), a 50 item, 11 subscale questionnaire used in graduate medical education in the Netherlands [11].

Educational evaluation can enhance professional practice and achieve the best medical education for trainees. It has been defined as a *“systematic approach to the collection analysis and interpretation of information about any aspect of the conceptualisation, design, implementation and utility of education programmes”* [12]. Well constructed evaluation is rigorous and defensible and has been linked to improvement in patient care [13].

**Figure 1:**
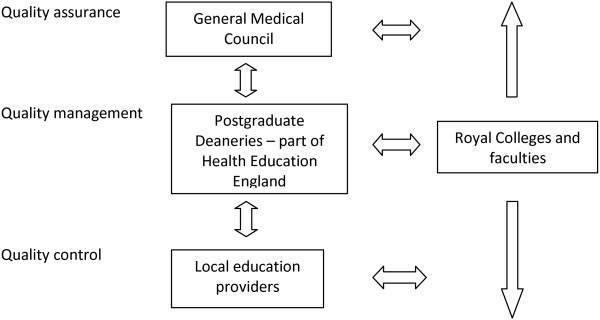
**Quality assurance, quality management and quality control of UK postgraduate medical training.**

**Figure 2:**
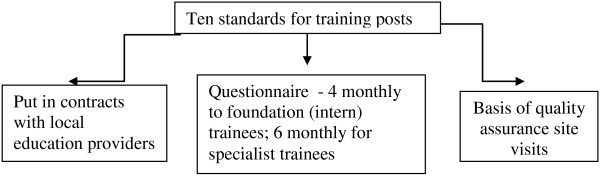
**West Midlands Deanery standards for evaluation of posts prior to 2006.**

The GMC has overarching responsibility for QA of medical education in the UK with the postgraduate deaneries managing the regional (QM) process (Figure 1) [14]. Health Education West Midlands (HEWM) has around 10% of UK trainees numbering over 5000 trainees in post. Prior to the GMC taking overarching control of QA, the West Midlands Deanery (now a part of HEWM) had drawn up ten standards for quality evaluation (Figure 2) [15, 16]. These were based on an earlier questionnaire with 9 standards, drawn up by extensive literature review and an expert panel, piloted and evaluation with collection of over 15,000 individual data sets. In 2006, an annual national UK trainees' survey was begun by the GMC using their standards for training posts and 9 domains for postgraduate deaneries (Table 1) [17, 18].

**Table 1:** **General Medical Council standards for Postgraduate Deaneries**

Domain Number	Domain
1	Patient safety
2	Quality management, review and evaluation
3	Equality and diversity and opportunity
4	Recruitment, selection and appointment*
5	Delivery of approved curriculum including assessment
6	Support and development of trainees, trainers and local faculty
7	Management of education and training
8	Educational resources and capacity
9	Outcomes (of training programmes)

*Domain 4 is not assessed by trainee survey but by collecting information regarding Deanery recruitment processes.

It is difficult to access detailed local information from the national GMC trainee survey especially in depth specialty and subspecialty information. This difficulty, subsequently supported by the three 2013 reports into quality and safety of the NHS [1–3] confirmed our belief that a simple local monitoring process, measuring good educational practice requires a local trainee questionnaire for each post in a training year. This together with trust self evaluation and monitoring visits would gather local quality assurance information that was blueprinted to standards as recommended by Dent and Harden [19].

The aim of this iterative study was i) to develop a new simple one page trainee questionnaire based on the GMC standards for postgraduate medical education ii) to look at the reliability and validity of the questionnaire as a tool for assessing postgraduate training posts with comparison to a well established and internationally validated tool, PHEEM.

## Methods

This work was undertaken between 2006 and 2011 and consists of the development of a tool followed by testing and validation using a 3 step process. The timeline for development and testing of JEST is shown in Table 2.

**Table 2:** **Timeline of development and testing of JEST**

Date	Progress
March to October 2006	JEST is developed with 15 standards based on the General Medical Council requirements
2007	Pilot testing of JEST by Foundation trainees
2008	Field testing of JEST by Paediatric specialist registrars
2008-2011	Widespread use of JEST by Foundation trainees

### Development of the new framework including the new fifteen standards

In March 2006, an expert group consisting of West Midlands associate deans, who are doctors working in postgraduate medical education, drew up the fifteen standards for training posts. These standards were based on the previously validated West Midlands questionnaire but also incorporating GMC standards on aspects of medical education including patient safety, evidence based practice and a junior doctors’ forum [18]. In common with tools on educational environment including the D-RECT one, this one page document, agreed in October 2006, included availability of senior doctor cover (educational supervision), appraisal and assessment, feedback, protected teaching (formal education), service based teaching (attending’s role) and input of the programme director (similar to the Dutch specialty tutor).

### The Fifteen Requirements for doctors and dentists in training posts in the West Midlands Deanery mapped onto the GMC standards

1. **Patient Safety** - All doctors and dentists in training must make patient safety their prime concern. All of the criteria below must be understood in terms of this overarching concept of patient safety.
2. **Programme Director** - each training programme must have a named programme director who accepts responsibility for planning the programme and ensuring that the standards set out below are met within the training programme.
3. **Induction** - at the beginning of each post, all trainees must attend induction programmes designed to familiarise them with both the Trust in general and the specialty department (organisational and educational aspects) in particular. Clinical guidelines used in the department must be explained at the induction. Written information on timetables and other arrangements must be provided. The induction must include details of occupational health services, arrangements in place to deal with bullying and harassment issues, and guidance in place and what to do in terms of whistle blowing in the NHS.
4. **Appraisal and Assessment** - Each trainee must have a named educational supervisor, who meets with him/her privately at the start of each attachment, and then at specified intervals to carry out appraisals, clarify career goals, identify learning needs and plan the education accordingly. Information from the consultant/trainer (if this is a different person from the educational supervisor) about the trainee's progress must be provided for these sessions. Appraisals and assessments must be properly documented using the specified documentation.
5. **Feedback** - The consultant/trainer must give regular helpful constructive feedback on performance in daily clinical supervision. All those involved in training must provide regular informal constructive feedback on both good and poor performance and contribute to appraisal and assessment of the trainees.
6. **Protected Teaching** - There must be a protected teaching programme for all trainees. This educational activity must be based on the relevant Royal College/Faculty curriculum, and separate from clinical work, and must be provided on a regular basis. Trainees must attend a minimum of 70% of these. The programme must be evaluated by the trainees and modified in the light of their feedback.
7. **Service Based Teaching** - arrangements must be in place, including arrangements for cross-specialty cover - if applicable.There must be opportunities to be taught and to learn during routine work, with appropriate consultant ward rounds, outpatient clinics and operating sessions per week. Handover.
8. **Senior Doctor Cover** - The immediate personal assistance of a senior doctor (normally a consultant or trainer) must always be available to trainees.
9. **Clinical Workload** - All trainees must be exposed to an appropriate level of clinical activity, to develop their clinical knowledge, skills and attitudes appropriate to their stage of educational development, and for the achievement of their educational objectives.
10. **Evidence Based Medicine and Audit** - Written guidelines on the management of common clinical conditions agreed locally in the specialty must be available to the trainees. These should be evidence based and subject to audit involving the trainees. All trainees must take an active part in audit and receive guidance and appropriate support to carry out this work.
11. **Inappropriate Tasks** - No trainee should be expected to perform work for which he/she is inadequately trained, which is of no relevance to his/her educational objectives, or which is prohibited by GMC/GDC guidelines (for example taking consent inappropriately).
12. **Rotas –** The rota must be compliant with current legislation, and monitored regularly to ensure that it remains compliant. Trainees must take part in the monitoring processes, when these occur.
13. **Accommodation and Catering** - The employer is responsible for the provision and maintenance of a safe working environment for the trainees, with accommodation and catering which meet current national standards.
14. **Leave** – All trainees must be allowed to undertake annual leave and study leave within their Terms and Conditions of Service. Study leave must be appropriate to their educational objectives, agreed with their educational supervisor in advance, and within the limits set by the regional postgraduate dean.
15. **Junior Doctors’ Forum** – There must be a junior doctors’ and dentists’ forum, which has representation from the employer, the educational supervisors and programme directors, and the trainees. This forum must meet **regularly**, and the meetings must be documented and minuted, including details of decisions made.

### Development of evaluation tools from these standards

**Figure 3:**
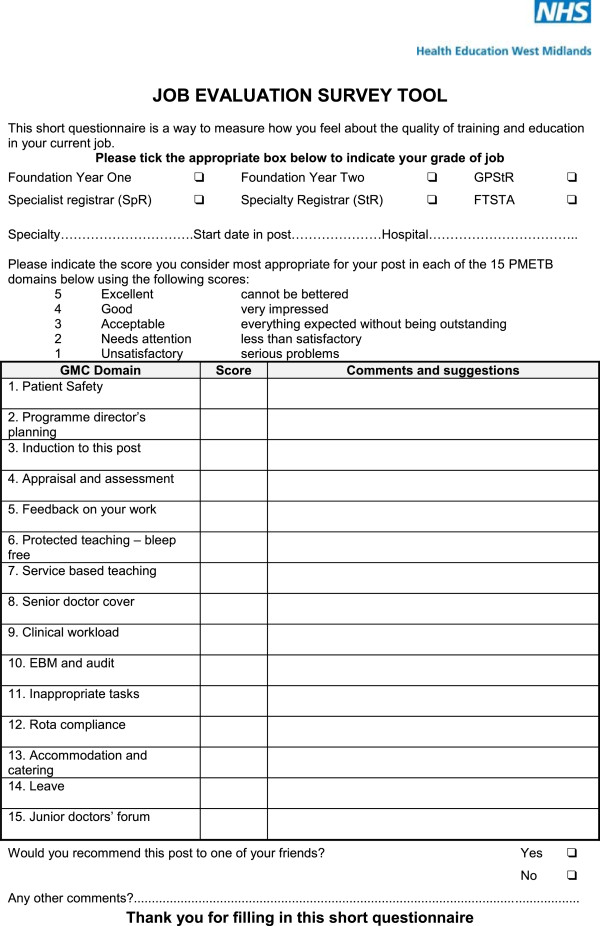
**The Job Evaluation Survey tool (JEST) questionnaire.**

The fifteen headings used in the standards were used to design a fifteen item evaluation form, the job evaluation survey tool (JEST) (Figure 3), to be sent to trainees at the end of each post to obtain their views about their posts. This was developed along the lines of our previous post evaluation form but with 15 categories instead of the previous ten [15, 16]. For each area there was a numerical score and space for free comments.

Validity was approached by basing the form on the well-established old post evaluation form [15, 16] and by basing the fifteen questions on the fifteen standards, derived from the GMC domains [18]. In addition, to ensure validity the results were compared with a well validated instrument, the Postgraduate Hospital Educational Environment Measure (PHEEM) [10] which has high reliability in many countries and different languages.

### Pilot testing of JEST in Foundation posts in two hospitals

In 2007, Foundation Year One and Foundation Year Two doctors (doctors in their first two years of postgraduate training) in 2 hospitals in the West Midlands Deanery (Walsall and Sandwell) were asked to complete the JEST trainee questionnaire to evaluate their current post. The questionnaire was handed out over a one month time period at weekly protected Foundation teaching which is compulsory and returned to the Postgraduate Centre Manager. Forms were anonymous.

### Field Testing in specialist registrar posts in Paediatrics in all hospitals in the West Midlands

In 2008, specialist paediatric registrars in all hospitals in the West Midlands Deanery were asked to complete the JEST trainee questionnaire to evaluate their current post. Questionnaires were handed out at monthly protected paediatric teaching which is compulsory with an email sent to all trainees asking them to complete the questionnaire if they were not at protected teaching due to night shifts or annual leave. The forms were anonymous and collected by the School of Paediatrics administrator.

### Widespread use by West Midlands Deanery Foundation doctors, comparing their completion of both JEST and PHEEM to evaluate their jobs

JEST and PHEEM were incorporated into the Foundation Programme ePortfolio by the West Midlands Deanery as an electronic questionnaire. Over a three year period (2008–2011), doctors in Foundation Years One and Two were asked to fill in both the JEST and the PHEEM at completion of each placement to evaluate their posts.

### Ethical considerations

Ethical approval was not required as this is a service evaluation study

(http://www.hra.nhs.uk/research-community/before-you-apply/determine-whether-your-study-is-research).

### Analysis

Data was coded into SPSS for both the JEST and PHEEM scores, demographic information and the response to the JEST question about recommending the post, namely *would you recommend this post to one of your friends? (yes or no)* (Figure 3*).* Statistical analysis using SPSS included mean scores, ranges, standard deviations, reliability using Cronbach’s alpha, correlations and factor analysis [20, 21]. Factor analysis was undertaken using principal component factor analysis with orthogonal (Varimax) rotation, accepting Eigen values over 1.0, and factor loadings over 0.5 [18].

In addition the scores were analysed using generalizability theory using the computer program GENOVA [22] including generalizability coefficient and variances of the various variables. Analyses were carried out to look at effect on response by a doctor’s gender, ethnicity, nationality and medical school.

## Results

### Foundation pilot

A total of 76 doctors out of 76 filled in and returned the JEST forms (100% return). Scores are shown in Table 3.

**Table 3:** **Scores for the JEST questions**

	Foundation doctors in pilot				Paediatric specialist registrars				Foundation doctors			
Domain	No	Mean scores	Score range	SD	No	Mean	Score range	SD	No	Mean	Score range	SD
**1. Patient safety**	75	3.85	2-5	0.82	170	3.77	2-5	0.74	3367	4.10	1-5	0.79
**2. Programme director's planning**	65	3.57	1-5	0.87	155	3.67	1-5	0.79	3367	3.92	1-5	0.76
**3. Induction to this post**	76	3.49	1-5	1.13	171	3.57	1-5	0.83	3367	3.89	1-5	0.90
**4. Appraisal and assessment**	76	3.93	1-5	0.81	159	3.74	1-5	0.70	3367	4.00	1-5	0.84
**5. Feedback on your work**	76	3.76	1-5	0.93	157	3.39	1-5	0.77	3367	3.86	1-5	0.93
**6. Protected teaching bleep free**	75	3.85	1-5	1.06	168	3.67	1-5	1.01	3367	3.92	1-5	1.02
**7. Service based teaching**	72	3.32	1-5	0.95	170	3.48	1-5	0.86	3367	3.77	1-5	0.93
**8. Senior doctor cover**	75	3.55	1-5	1.17	170	3.90	1-5	0.83	3367	3.95	1-5	1.00
**9. Clinical workload**	76	3.62	1-5	0.97	171	3.73	2-5	0.64	3367	3.77	1-5	0.90
**10. EBM and Audit**	75	3.61	1-5	0.82	153	3.64	1-5	0.70	3367	3.86	1-5	0.77
**11. Inappropriate tasks**	74	3.58	2-5	0.86	152	3.46	1-5	0.80	3367	3.82	1-5	0.86
**12. Rota compliance**	75	3.73	2-5	0.76	164	3.48	1-5	0.93	3367	3.75	1-5	.10
**13. Accommodation and catering**	61	3.51	1-5	0.91	144	3.02	1-5	0.94	3367	3.33	1-5	0.97
**14. Leave**	76	3.63	1-5	1.08	168	3.56	1-5	0.86	3367	3.91	1-5	0.91
**15. Junior doctors' forum**	52	3.58	2-5	0.75	111	3.00	1-5	0.94	3367	3.69	1-5	0.79
**Total JEST score**									3367	57.57	75.0	8.87

### Overall scores

For overall scores, most of the 76 Foundation Doctors were able to answer all the questions, except for the questions on accommodation, catering and the junior doctors’ forum. The highest scores were given for appraisal and assessment, followed closely by patient safety and protected teaching (equal second highest). The lowest score was for service based teaching. The mean scores for all fifteen questions were above 3, the acceptable level.

#### Reliability

Reliability overall was very good at 0.91 using Cronbach’s alpha. Using the alpha if item deleted, there did not appear to be any rogue questions in the fifteen JEST form questions. Using generalizability theory and a simple P × I design the generalizability coefficient was 0.89, again a very high value. The variances were 0.30 for persons (the 76 doctors), 0.02 for items (the fifteen questions) and 0.53 for persons × items. This shows that there is a greater variance between what the doctors think of their jobs, rather than between the fifteen questions.

### Field testing in specialist registrar posts in Paediatrics in all hospitals in the West Midlands (Table 3)

A total of 173 specialist registrars out of 173 in paediatrics from 17 hospitals in the West Midlands filled in and returned the JEST form (100% return).

Descriptive statistics showed the highest score was for senior doctor cover, followed by patient safety and assessment and appraisal. The lowest score was for the junior doctors’ forum. In terms of reliability, the overall reliability using Cronbach’s alpha was 0.88. There were no rogue questions using the alpha if item deleted function. A generalizability study showed a generalizability coefficient of 0.86. The error variances were persons 0.18, items (the 15 questions) 0.06 and persons × Items 0.44.

### Comprehensive use of JEST by West Midlands Deanery foundation doctors including comparison of JEST and PHEEM in the evaluation of posts

There were 3367 responses consisting of 51% FY1 doctors, 41% males, 59% Caucasian background, 91% British nationals, 92% UK Medical School graduates and 2% (69) who reported themselves as having a disability. In all 3367 responders, there were scores for the JEST (Table 3) and for the PHEEM.

**Figure 4:**
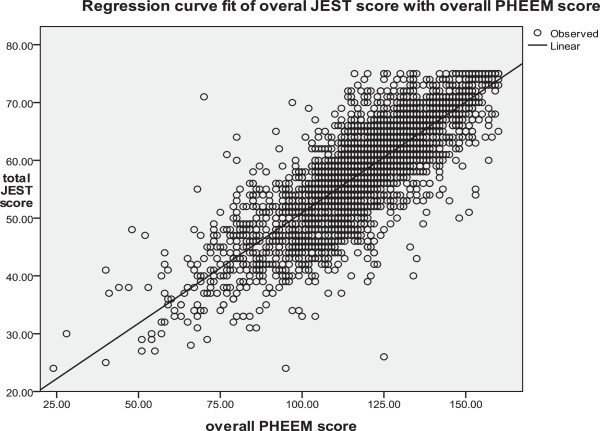
**Correlation between overall PHEEM scores and total Jest Scores for 3367 sets of evaluation scores.**

Reliability for the 3367 sets of JEST results revealed a Cronbach’s alpha of 0.91 with no rogue questions using the alpha if item deleted function. The overall score for PHEEM and scores for three subscales are shown in Table 4. The correlation between the total JEST scores and the overall PHEEM scores was 0.80 using the Spearman’s correlation (as both are Likert scale ordinal data) (Figure 4) (p < 0.001).

**Table 4:** **Descriptive Statistics for PHEEM overall and three subscales**

Domain	Number	Minimum	Maximum	Mean	Standard deviation
**Overall PHEEM score**	3367	24.00	160.00	117.31	18.82
**Role autonomy subscale**	3367	8.00	56.00	40.53	6.98
**Teaching subscale**	3367	1.00	60.00	45.26	8.59
**Social subscale**	3367	9.00	44.00	31.52	4.80

Factor analysis (Table 5) for the 3367 sets of JEST data showed Eigenvalues for the 15 questions 43.03 to 1.09 i.e. all were greater than 1 with two factors produced after three iterations, responsible for 53% of the total variance. The Kaiser Meyer Olkin test had a value of 0.94, and Bartlett’s test was highly significant, indicating a very high level of sampling adequacy. Looking at the composition of these two factors, factor one is about teaching and factor two is about social and lifestyle issues. In fact, correlations of these two factors with the three PHEEM subscales supported this, with factor one most strongly correlated with the PHEEM teaching subscale (Spearman’s correlation 0.7, p < 0.001) and factor two most strongly correlated with the social subscale (Spearman’s correlation 0.47, p < 0.001).

**Table 5:** **Factors from 3367 Foundation doctors JEST scores**

JEST questions	Factor	
	1	2
**5. Feedback on your work**	0.815	
**4. Appraisal and assessment**	0.793	
**7. Service based teaching**	0.688	
**2. Programme director's planning**	0.675	
**3. Induction to this post**	0.661	
**1. Patient Safety**	0.629	
**8. Senior doctor cover**	0.593	
**10. EBM and Audit**	0.503	
**14. Leave**		0.740
**13. Accommodation and catering**		0.688
**12. Rota compliance**		0.688
**15. Junior doctors' forum**		0.632
**9. Clinical workload**	0.512	0.519
**11. Inappropriate tasks**		0.519
**6. Protected teaching - bleep free**		

Looking at the global question of “*would you recommend this post to one of your friends*”, 2958 (88%) replied yes and 409 (12%) replied no. The differences in the total JEST score between those who replied yes and no was highly significant (P < 0.001) using a Mann Whitney test. There was no difference in response to the JEST questions by a doctor’s gender (Mann Whitney p = 0.92) or nationality (Kruskal Wallis p = 0.33). Looking at medical school, international graduates had significantly higher JEST scores (Kruskal Wallis p = 0.03) with respect to ethnicity. Afro-Caribbean doctors gave significantly lower scores (Kruskal Wallis p = 0.03).

## Discussion

The Job Evaluation Survey Tool (JEST), consisting of 15 questions was developed against GMC standards, and evaluated in a pilot study, field testing and finally in comprehensive use in the Foundation programme. It had high reliability (Cronbach’s alpha 0.88-0.91 and generalizability coefficient of 0.86-0.89). The validity of the tool was established by building on the previous 10 item version of the West Midlands Deanery post evaluation tool [15, 16]. Concurrent validity measured against PHEEM was high.

JEST measures many similar attributes to the well validated PHEEM [10] and D-RECT [11], but it is a much simpler one page questionnaire with only 15 questions and a final overall question about recommending the job, compared to 40 questions in PHEEM and 50 in D-RECT. It is therefore much easier to use and quicker to complete than PHEEM and D-RECT and is available in electronic format. We continue to use PHEEM if JEST raises concerns and there is a need to look in more depth at posts. JEST is now completed by all West Midlands trainees at the end of their 4 and 6 month posts. The high trainee completion rate of JEST seen in this study has continued and the fact that it is contained on one side of A4 and is simple and easy to complete are most likely contributing factors to this high response rate. Another contributing factor is likely to be the difference that completion of JEST has made to training posts enabling a continuous cycle of improvement and issues addressed in a timely manner. In one or two cases, removal of training posts from a hospital trust has resulted if this cannot be achieved by the Deanery working with the Trust and QA visits led by the Postgraduate Dean.

The high reliability of JEST (Cronbach’s alpha 0.89-0.91) compares well with values achieved in the GMC 2006 trainees survey, where results varied from 0.36 to 0.89 (scores were 0.41. 0.80, 0.72, 0.78, 0.36, 0.89. 0.54, 0.47 and 0.63 for the various parts of the questionnaire) [23]. In further surveys, it has been difficult to find these values.

Limitations of this study include the fact that JEST was formally evaluated in one Deanery only (although it is used in other Deanery Schools of Medicine) and has only been used in the UK. The fact that there was no difference in responses to the questions by gender or nationality supports its use nationwide. These results also support the use of JEST in other countries. Other limitations of the study include it being tested in Paediatrics only rather than a wide range of specialties. JEST has however subsequently been used in all specialties in the West Midlands Deanery and found to be a useful tool across all specialties including those which are hospital based and community ones such as general practice and public health. The authors of this study consist of senior clinicians and a senior manager. As questionnaires were distributed by postgraduate centre managers and the school of Paediatrics administrator and completed anonymously we do not feel that the positions of power of the authors compared to the trainees affected the results of the study. The West Midlands has had a culture of encouraging reporting of concerns about posts and for the Deanery to act upon those concerns to improve training. We were fortunate to get excellent returns of the questionnaires in pilot and field testing despite not making return compulsory.

The JEST has become an integral part of quality management in the West Midlands Deanery and is used in conjunction with the GMC annual trainees’ survey and trust self reporting for the annual deanery report. Monitoring visits to Trusts are triggered if JEST forms completed by trainees identify consistently poorly regarded posts, especially in the JEST categories of *patient safety, feedback, service based teaching* and *clinical workload.*

Work in progress is sharing the JEST tool with other Deaneries in the UK as well as potential international collaboration. It will be important to revisit our 15 standards and thus the JEST proforma if there are any changes in GMC emphasis of aspects of medical education. This could well include requiring additional standards in the future and adding to the number of domains in JEST. It will be important to ensure that the simplicity and ease of completion of this tool is not compromised if changes are made.

## Conclusion

We recommend the JEST as a one page questionnaire which is simple, valid and reliable, as a means of quality assuring posts in postgraduate medical training. Jones et al. described quality assurance in European dental schools and the importance of having a toolkit, with selection of the right evaluation tools to suit each environment [24]. We recommend JEST for that toolkit and in particularly to ensure that trainees’ views on their training programmes are sought, in keeping with recommendations from reports into patient safety in the NHS [1–3]. Quality assurance and quality improvement of training programmes apply worldwide. With local adaptations, this simple one page questionnaire could be used to collect information on training posts from postgraduate medical and dental trainees not only in the UK but also internationally.

## Authors’ information

David Wall MB ChB, MMEd, PhD, FRCP, is retired from his post as deputy regional postgraduate dean and professor of medical education in the West Midlands Deanery, Birmingham, UK. He is now tutor in medical education at the Centre for Medical Education, University of Dundee.

Helen Goodyear MB ChB, MMEd, MD, FRCP, FRCPCH, MA is a Consultant Paediatrician at Heart of England NHS foundation Trust, Head of the Postgraduate School of Paediatrics and an Associate Postgraduate Dean at Health Education West Midlands, UK.

Baldev Singh MB BS, MD, FRCP is Foundation Dean for the Black Country Foundation School and a consultant physician at New Cross Hospital in Wolverhampton, UK.

Andrew Whitehouse MA, MB BChir, FRCP is Head of Postgraduate School of Medicine and Foundation Programme Director for Health Education West Midlands, UK.

Elizabeth Hughes BSc MB ChB, FRCP, is postgraduate medical dean at Health Education West Midlands and professor in chemical pathology at Sandwell and West Birmingham Hospitals NHS Trust in the West Midlands, UK.

Jonathan Howes BA, MSc is Information Manager for Health Education West Midlands, UK.
